# Prognostic Significance of EZH2 Expression in Non-Small Cell Lung Cancer: A Meta-analysis

**DOI:** 10.1038/srep19239

**Published:** 2016-01-12

**Authors:** Xinhua Wang, Hongqing Zhao, Lei Lv, Liang Bao, Xun Wang, Shuguang Han

**Affiliations:** 1 Department of Respiratory Medicine, Nanjing Medical University Affiliated Wuxi Second Hospital, Wuxi 214002, Jiangsu Province, China

## Abstract

Various studies examined the relationship between EZH2 overexpression with the clinical outcome in patients with non-small cell lung cancer (NSCLC), but yielded inconsistent results. Electronic databases updated to Dec 2014 were searched to find relevant studies. A meta-analysis was conducted with eligible studies which quantitatively evaluated the relationship between EZH2 overexpression and survival of patients with NSCLC Survival data were aggregated and quantitatively analyzed. We performed a meta-analysis of 10 studies (n = 1,695 patients) that evaluated the correlation between EZH2 overexpression and survival in patients with lung cancer. Combined hazard ratios suggested that EZH2 overexpression was associated with poor prognosis of overall survival (OS) (HR = 1.68, 95% CI: 1.42–1.93) in patients with lung cancer. In the stratified analysis, significantly risks were found among Asians (HR = 1.33, 95% CI: 1.62–1.70), lung adenocarcinoma patients (HR = 1.75, 95% CI: 1.38–2.52, in stage I NSCLC patients (HR = 2.51, 95% CI: 1.23–3.79), but not among Caucasians. EZH2 overexpression indicates a poor prognosis for patients with NSCLC, this effect appears also significant when the analysis is restricted in Asian population, lung AC and stage I patients, but not among Caucasians.

Lung cancer is a leading cause of cancer deaths in the world. Approximately 80–85% of all lung cancers are non-small-cell lung cancer (NSCLC)^1^. The prognosis for lung cancer patients is generally poor, with an overall 5 year survival rate of approximately 15%, and it has shown little improvement in recent decades^2^,^3^. Several independent prognostic factors for survival have been identified: performance status (PS), disease stage, age, sex and amount of weight lost^4^. Some of these factors are useful when choosing treatment options for an individual, principally disease stage and PS. However, the discriminant value of most potential prognostic biological markers is insufficient to predict the optimal therapeutic course for an individual^5^.

Enhancer of zeste homolog 2 (EZH2) is a key component of the polycomb repressive complex 2, which possesses histone methyltransferase activity and mediates gene silencing through posttranslational histone modifications^6^. EZH2 is frequently overexpressed in a wide variety of human malignancies such as breast cancer^7^, prostate cancer^8^, gastric cancer^9^, colorectal cancer^10^ and lung cancer. In addition, it also promotes cancer development and progression through chromatin modification by epigenetic activation of oncogenic signaling cascades and silencing of tumor suppressor genes, and has been implicated in cell proliferation, differentiation, invasion, and metastasis^11^. Thus, it is acting with oncogenic properties.

Many studies have evaluated whether the overexpression of EZH2 may be a prognostic factor for survival in patients with lung cancer. However, the results of the studies are inconclusive and no consensus has been reached. It is unknown whether differences in these investigations have been mostly due to their limited sample size or genuine heterogeneity. Thus, we conducted a meta-analysis of all available studies relating EZH2 with the clinical outcome in patients with lung cancer.

## Materials and Methods

### Search strategy and study selection

The electronic databases PubMed, Embase and CNKI (China National Knowledge Infrastructure) were searched for studies to include in the present meta-analysis. An upper date limit of Dec 01, 2014 was applied; we used no lower date limit. Searches included the terms “lung”, “cancer or carcinoma or tumour or neoplasm”, “EZH2”, “Enhancer of zeste homolog 2”, and “prognosis”. We also reviewed the Cochrane Library for relevant articles. The references reported in the identified studies were also used to complete the search.

Studies eligible for inclusion in this meta-analysis met the following criteria: (1) measure EZH2 expression in the primary lung cancer with IHC (immunohistochemistry) or Realtime-PCR (polymerase chain reaction); (2) provide information on survival (i.e. overall survival [OS], studies investigating response rates only were excluded); (3) When the same author reported results obtained from the same patient population in more than one publication, only the most recent report, or the most complete one, was included in the analysis. Two reviewers (X.W. and H.Z.) independently determined study eligibility. Disagreements were resolved by consensus.

### Data extraction and quality assessment

The final articles included were assessed independently by two reviewers (X.W. and H.Z.). Data retrieved from the reports included author, publication year, patient source, histology, study design, test method, positive, follow-up and survival data (Table 1). If data from any of the above categories were not reported in the primary study, items were treated as “not applicable”. Only four studies did not provide the data of positive of EZH2, which did not affect the subsequent statistical analysis. Thus, we did no contact the author of the primary study to request the information. We did not use prespecified quality-related inclusion or exclusion criteria and did not weigh each study by a quality score, because the quality score has not received general agreement for use in a meta-analysis, especially observational studies^12^.

### Statistical methods

For the quantitative aggregation of the survival results, we measured the impact of EZH2 overexpression on survival by HR between the two survival distributions. HRs and 95% confidence intervals (CIs) were used to combine as the effective value. If the HRs and their 95% CIs were given explicitly in the articles, we used crude ones. When these variables were not given explicitly, they were calculated from the available numerical data using methods reported by Parmar *et al.*^13^.

Heterogeneity of the individual HRs was calculated with χ^2^ tests according to Peto’s method^14^. Heterogeneity test with inconsistency index (*Ι*^2^) statistic and *Q* statistic was performed. If HRs were found to have fine homogeneity, a fixed effect model was used for secondary analysis; if not, a random-effect model was used. DerSimonian-Laird random effects analysis^15^ was used to estimate the effect of EZH2 overexpression on survival. By convention, an observed HR > 1 implies worse survival for the group with EZH2 overexpression. The impact of VEGF on survival was considered to be statistically significant if the 95% confidence interval (CI) did not overlap with 1. Horizontal lines represent 95% CIs. Each box represents the HR point estimate, and its area is proportional to the weight of the study. The diamond (and broken line) represents the overall summary estimate, with CI represented by its width. The unbroken vertical line is set at the null value (HR = 1.0).

Evidence of publication bias was sought using the methods of Egger *et al.*^16^ and of Begg *et al.*^17^. Intercept significance was determined by the *t* test suggested by Egger (*P* < 0.05 was considered representative of statistically significant publication bias). All of the calculations were performed by STATA version 11.0 (Stata Corporation, College Station, TX).

## Results

### Study selection and characteristics

Ten studies^18^,^19^,^20^,^21^,^22^,^23^,^24^,^25^,^26^ published between 2010 and 2014 were eligible for this meta-analysis. All reported the prognostic value of EZH2 status for survival in lung cancer patients. The total number of patients included was 1695, ranging from 83 to 360 patients per study (median 169). The major characteristics of the 10 eligible publications are reported in Table 1. The studies were conducted in 3 countries (China, USA and Japan). Among the 10 studies, 6 studies (1041 patients, 61.4%) were performed in Asian populations, and the remaining 4 studies (654 patients, 38.6%) followed Non-Asian patients. All patients in the eligible studies were determined by pathological stage.

All of the studies reported the prognostic value of EZH2 status for survival in patients with lung cancer. All of the 10 studies reported HRs (multivariate analysis) explicitly. A HR on OS could be extracted for 10 studies. Eight of the 10 studies identified EZH2 overexpression as an indicator of poor OS, and the other 2 studies showed no statistically significant impact of EZH2 overexpression on OS.

### Meta-Analysis

**Figure 1:**
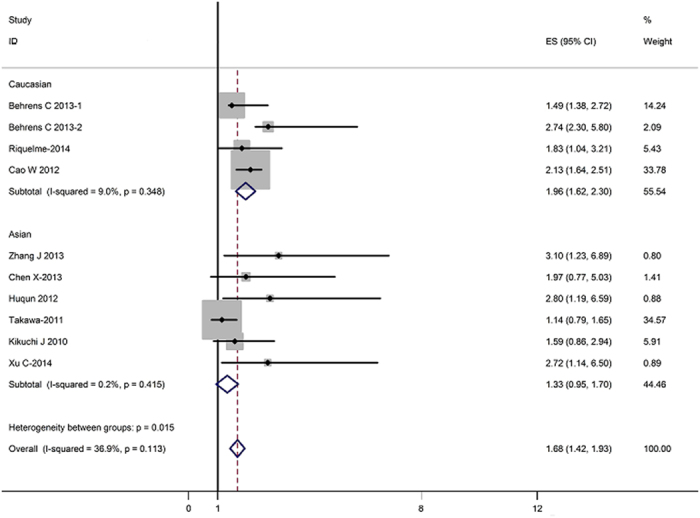
Meta-analysis (Forest plot) of the 10 evaluable studies assessing EZH2 in lung cancer stratified by patient source for overall survival.

The results of the meta-analysis were shown in Table 2 and Fig. 1. Overall, the combined HR for all 10 eligible studies evaluating EZH2 overexpression on OS was 1.68(1.42–1.93), suggesting that EZH2 overexpression was associated with poor prognosis for lung cancer. No significant heterogeneity was observed among the studies (*Q* = 7.39, *I*^2^ = 36.9%, *P* = 0.113). When grouped according to the geographic settings of individual studies, the combined HRs of Asian studies and Caucasian studies were 1.33(1.62–1.70) and 1.96(0.42–2.30), respectively, indicating EZH2 was an indicator of poor prognosis in Asian populations but not for Caucasians.

**Figure 2:**
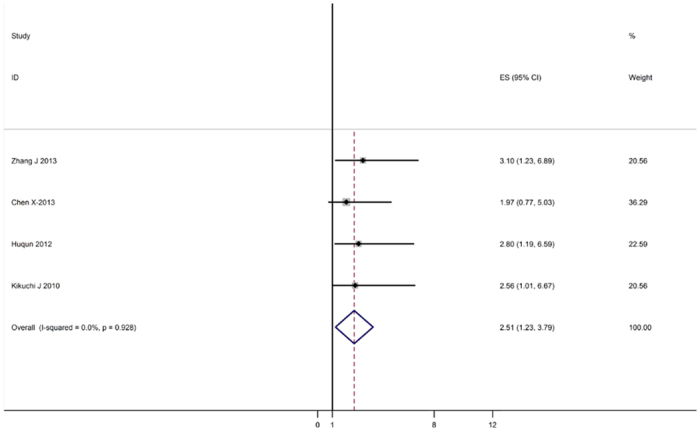
Meta-analysis (Forest plot) of the 4 evaluable studies assessing EZH2 in lung cancer stratified by patient source for overall survival.

The data were adequate to aggregate the studies of lung adenocarcinoma and stage I NSCLC for subgroup analyses. When we aggregated three studies that reported results for adenocarcinoma, the combined HR was statistically significant: HR 1.75 (95% CI: 1.38–2.52, I^2^ = 46.2%, P = 0.154 for heterogeneity). We also observed a statistically significant effect of EZH2 expression on survival in stage I patients with an HR of 2.51 (95% CI: 1.23–3.79, I^2^ = 0%, P = 0.928 for heterogeneity) (Fig. 2).

### Publication Bias

**Figure 3:**
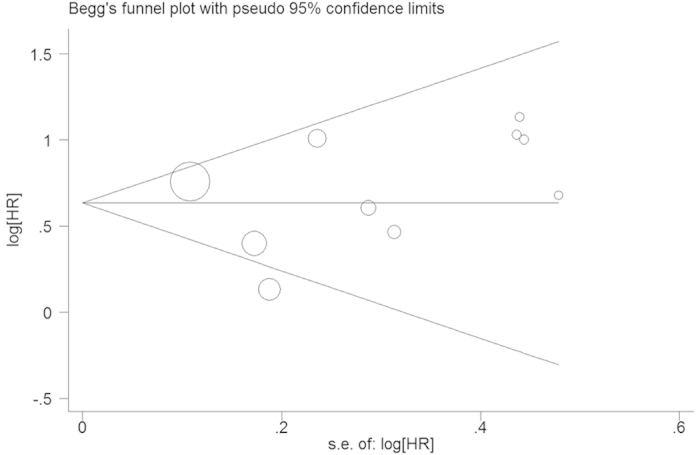
Funnel plot of the 10 evaluable studies assessing EZH2 in lung cancer for overall survival.

**Figure 4:**
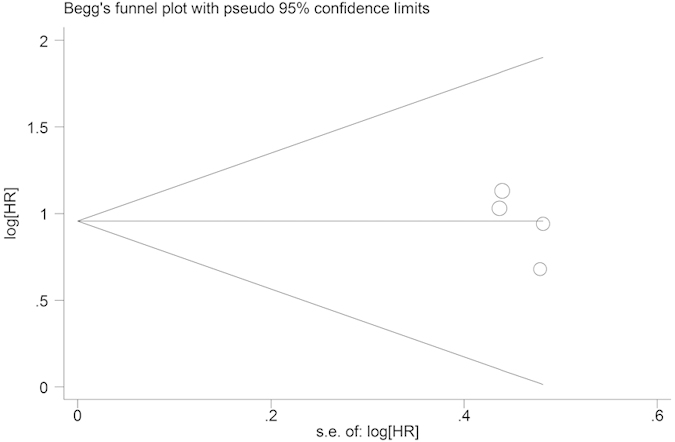
Funnel plot of the 4 evaluable studies assessing EZH2 in stage I lung cancer for overall survival.

Begg’s funnel plot and Egger’s test were performed to assess the publication bias in the literature. All 10 eligible studies investigating EZH2 overexpression on overall survival yielded a Begg’s test score of p = 0.260 and an Egger’s test score of p = 0.620, meanwhile according to the funnel plot (Fig. 3), the absence of publication bias was found. Similar results were found for investigating EZH2 overexpression on overall survival of stage I NSCLC patients (a Begg’s test score of p = 0.774 and an Egger’s test score of p = 0.221) (Fig. 4). These results suggested that there were no publication biases in these subgroup analyses.

## Discussion

EZH2 is the catalytic subunit of PRC2, which catalyses the addition of methyl groups to lysine 27 on histone H3 (H3K27) in the promoters of target genes, leading to repression of gene transcription^27^,^28^. The gene of EZH2, encoding a polycomb group protein, plays an important role in tumorigenesis and cancer progression through epigenetic gene silencing and chromatin remodeling^29^.

Our present meta-analysis is the first to evaluate the correlation between EZH2 overexpression and survival in patients with lung cancer. The meta analysis combined 10 publications including 1,695 patients with lung cancer to yield statistics, indicating statistically significant role of EZH2 on overall survival in lung cancer. Combined hazard ratios suggested that EZH2 overexpression was associated with poor prognosis of overall survival (OS) (HR = 1.68, 95% CI: 1.42–1.93) in patients with lung cancer. In the stratified analysis, significantly risks were found among Asians (HR = 1.33, 95% CI: 1.62–1.70), lung adenocarcinoma patients (HR = 1.75, 95% CI: 1.38–2.52, in stage I NSCLC patients (HR = 2.51, 95% CI: 1.23–3.79), but not among Caucasians (HR = 1.96, 95% CI: 0.42–2.30). When analysis was restricted to stage I lung cancer, we found that the combined HR (2.51) was larger than the combined HR for all 10 eligible studies of stages I-III (1.68), suggesting that EZH2 expression could be an important prognostic factor for early-stage lung cancer.

The heterogeneity issue was complicated in the systematic review and meta-analysis was. We found no significant heterogeneity among all studies included and subgroup analysis. Another potential source of bias is related to the method of HR and 95% CI extrapolation. If these statistics were not reported by the authors, we calculated them from the data available in the article. If this was not possible, we extrapolated them from the survival curves, necessarily making assumptions about the censoring process. Data for multivariate survival analysis reported in the article were included in the present systematic review with meta-analysis; if these data were not available, data calculated from survival curves by univariate analysis were included. These results should be confirmed by an adequately designed prospective study. Furthermore, the exact value of EZH2 overexpression status needs to be determined by appropriate multivariate analysis. Unfortunately, few prospectively designed prognostic studies concerning biomarkers have been reported; thus, our collection of many retrospective studies revealed more significance.

Our data were not consistent with the results of a previous meta-analysis^30^ included four studies with gastric cancer patients, that meta-analysis results demonstrated that the OS of EZH2-negative patients was shorter than that of patients with positive expression of EZH2 (HR = 0.54, 95% CI = 0.05–1.03). The previous meta-analysis included only 4 Asian studies, and the data were insufficient to determine the prognostic value of EZH2 for subgroups divided according to histology or disease stage. In our study, however, we have focused on the prognostic significance of EZH2 in NSCLC patients. We performed this systematic review and meta-analysis to yield summary statistics by including more recent related studies and by generally using a more comprehensive search strategy. Screening, study selection, and quality assessment were performed. We also explored heterogeneity and potential publication bias in accordance with published guidelines.

Publication bias^31^ is a major concern for all forms of meta-analysis; positive results tend to be accepted by journals, while negative results are often rejected or not even submitted. The present analysis does not support publication bias; the obtained summary statistics likely approximate the actual average. However, it should be noted that our meta-analysis could not completely exclude biases. For example, the study was restricted to papers published in English and Chinese, which probably introduced bias.

In conclusion, despite the limitations described above, our meta-analysis is the first study to systematically estimate the association between EZH2 expression detected by IHC or Realtime-PCR and lung cancer survival. As determined in our meta-analysis, we concluded that EZH2 overexpression was associated with poor overall survival in lung cancer, this effect appears also significant when the analysis is restricted in Asian population, lung AC and stage I patients, but not among Caucasians. To strengthen our findings, well-designed prospective studies with better standardized assessment of prognostic markers should help to explore the relation between EZH2 overexpression and survival of lung cancer.

## Additional Information

**How to cite this article**: Wang, X. *et al.* Prognostic Significance of EZH2 Expression in Non-Small Cell Lung Cancer: A Meta-analysis. *Sci. Rep.* **6**, 19239; doi: 10.1038/srep19239 (2016).

## Figures and Tables

**Table 1:** Main characteristics and results of the eligible studies.

First author-year	Patients source	Histology	stage	N pts	Method	Positive (%)	covariates of multivariate analysis	HR estimation	Survival results
Behrens C 2013-set 1	USA	AC	I-III	320	IHC	NA	Age, gender, race, smoking status, disease stage	HR and 95% CI 1.49(1.38–2.72)	Poor
Behrens C 2013-set 2	USA	AC	I-III	91	IHC	NA	Age, gender, race, disease stage	HR and 95% CI 2.74(2.30–5.80)	Poor
Zhang J 2013	China	NSCLC	I	84	IHC	65.5	Age, gender, race, histology, smoking status	HR and 95% CI 3.10(1.23–6.89)	Poor
Chen X-2013	Japan	NSCLC	I	42	IHC	56	Age, gender, race, histology	HR and 95% CI 1.97(0.77–5.03)	NS
Huqun 2012	Japan	NSCLC	I	106	IHC	58.5	Age, gender, race, smoking status, histology	HR and 95% CI 2.80(1.19–6.59)	Poor
Takawa-2011	Japan	NSCLC	I-III	292	IHC	46.2	Age, gender, race, histology, disease stage	HR and 95% CI 1.14(0.79–1.65)	NS
Kikuchi J 2010	Japan	NSCLC	I-IV	157	IHC	NA	Age, gender, race, histology, smoking status, disease stage	HR and 95% CI 1.59(0.86–2.94)	NS
	Japan	NSCLC	I	83	IHC	NA	Age, gender, race, histology	HR and 95% CI 2.56(1.01–6.67)	Poor
Xu C-2014	China	NSCLC	IIIB- IV	360	IHC	56.7	Age, gender, race, histology, smoking status	HR and 95% CI 2.72(1.14–6.50)	Poor
Riquelme-2014	USA	AC	I-III	149	RealTime-PCR	49	Age, gender, race, disease stage	HR and 95% CI 1.83(1.04–3.21)	Poor
Cao W 2012	USA	NSCLC	I-IV	94	RealTime-PCR	50	Age, gender, race, histology, disease stage	HR and 95% CI 2.13(1.64–2.51)	Poor

IHC, immunohistochemistry; NSCLC, non-small-cell lung cancer; AC, adenocarcinoma; NS, not significant; NA: not applicable; HR, hazard ratio; N pts, number of patients; PCR, polymerase chain reaction.

**Table 2:** Meta-analysis: HR value in lung cancer subgroups according to histology and stage.

	Nb	Patients	Combined HR (95% CI)	χ^2^heterogeneity test (P)
Overall	10	1695	1.68(1.42–1.93)	0.113
Asian	6	1041	1.33(1.62–1.70)	0.415
Caucasian	4	654	1.96(0.42–2.30)	0.348
Adenocarcinoma	3	560	1.75(1.38–2.52)	0.134
Stage I	4	315	2.51(1.23–3.79)	0.928

Abbreviations: HR: hazard ratio; Nb: number of studies; CIs: confidence intervals.
